# Resolving Conflicts between Agriculture and the Natural Environment

**DOI:** 10.1371/journal.pbio.1002242

**Published:** 2015-09-09

**Authors:** Andrew J. Tanentzap, Anthony Lamb, Susan Walker, Andrew Farmer

**Affiliations:** 1 Ecosystems and Global Change Group, Department of Plant Sciences, University of Cambridge, Cambridge, United Kingdom; 2 Conservation Science Group, Department of Zoology, University of Cambridge, Cambridge, United Kingdom; 3 Landcare Research, Dunedin, New Zealand; 4 Institute for European Environmental Policy, London, United Kingdom

## Abstract

Agriculture dominates the planet. Yet it has many environmental costs that are unsustainable, especially as global food demand rises. Here, we evaluate ways in which different parts of the world are succeeding in their attempts to resolve conflict between agriculture and wild nature. We envision that coordinated global action in conserving land most sensitive to agricultural activities and policies that internalise the environmental costs of agriculture are needed to deliver a more sustainable future.

## Abstract 2

Agriculture dominates the planet but has many environmental costs that will escalate with global food demand. This Essay outlines a vision for a more sustainable future.

## Introduction

**Fig 1:**
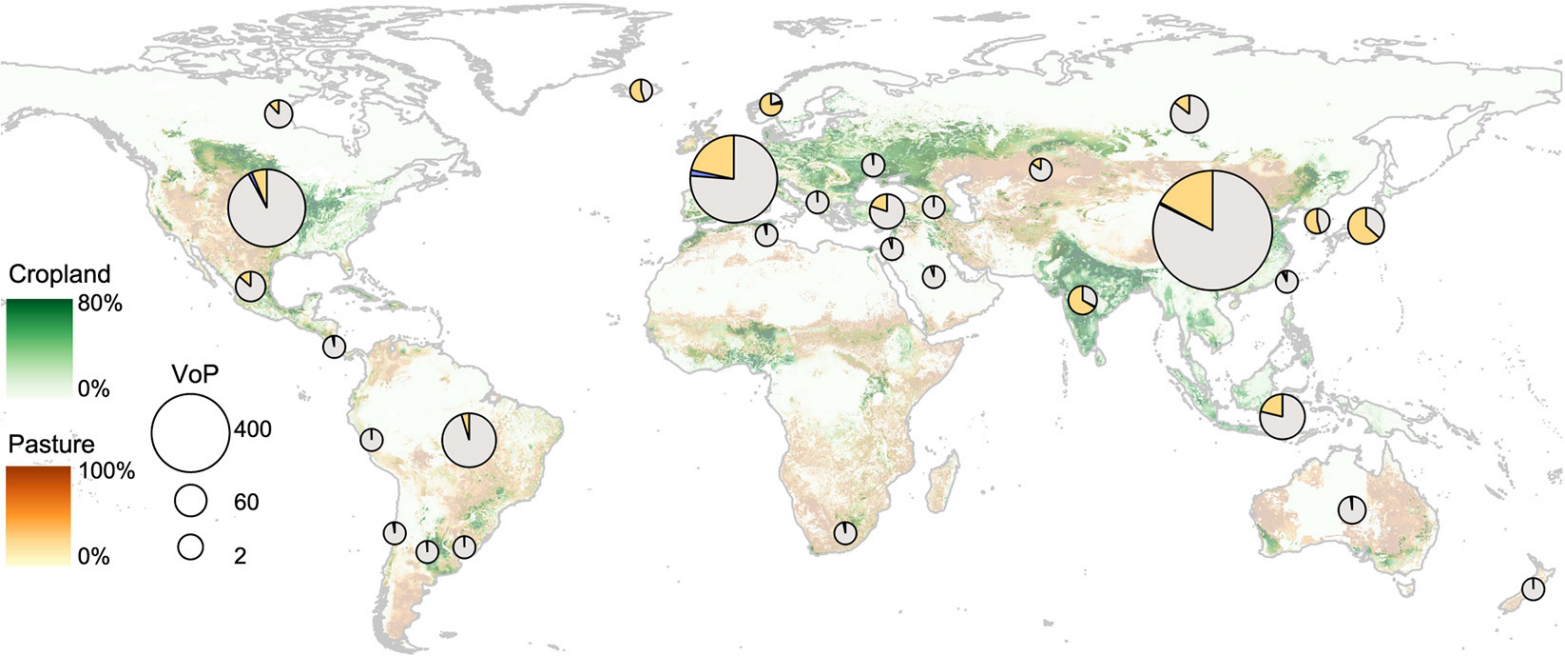
Financial support to farmers from taxpayers and consumers associated with agricultural policies as a proportion of the total value of agricultural production (VoP) at the farm gate. We distinguished between policies aiming to achieve specific environmental objectives beyond those required by regulation (purple segments) and all other types of support irrespective of their influence on farm production or income (orange segments), e.g., market price support, monetary transfers based on output, input subsidies. Sizes of symbols were scaled to VoP in hundreds of millions of United States dollars. Data are from the most recent year available (2008–2013) and described in S1 Text. Green and brown shading in the background is the percent of cropland and pasture at 5’ resolution in the year 2000 [7]. Financial support data were preferentially sourced from the Organisation for Economic Cooperation and Development (OECD) (*n* = 21 countries), supplemented from the World Trade Organization (WTO) where possible for additional countries (*n* = 12). Data used to make this figure are provided in S1 Data.

Agriculture is the most dominant land use on Earth and will remain so as the world’s population and global food demand rise [1] (Fig 1). This poses a fundamental challenge to the natural environment, which requires access to many of the same resources as agricultural producers and is harmed by agriculture’s external impacts [2,3]. Using land resources for agriculture prioritises crop and livestock production over the many other benefits that natural ecosystems provide and that agriculture displaces, including biodiversity, carbon storage, and water purification [4]. Governments also heavily support agricultural production in ways that are often bad for the environment, such as rewarding output or lowering input prices [5,6]. Very little is spent on actively mitigating the environmental impacts (Fig 1).

Here, we summarise how agriculture impacts the natural environment and then scrutinise the success of government agricultural policies that we believe offer the most promise for safeguarding nature while feeding a growing human population. We use economic and political theory to help explain why some measures are more prone to succeed than others, recognising that scientific understanding of a problem does not mean it will be solved [8]. Throughout, we touch upon examples from centres of agricultural production.

## A Cultivated Planet

**Fig 2:**
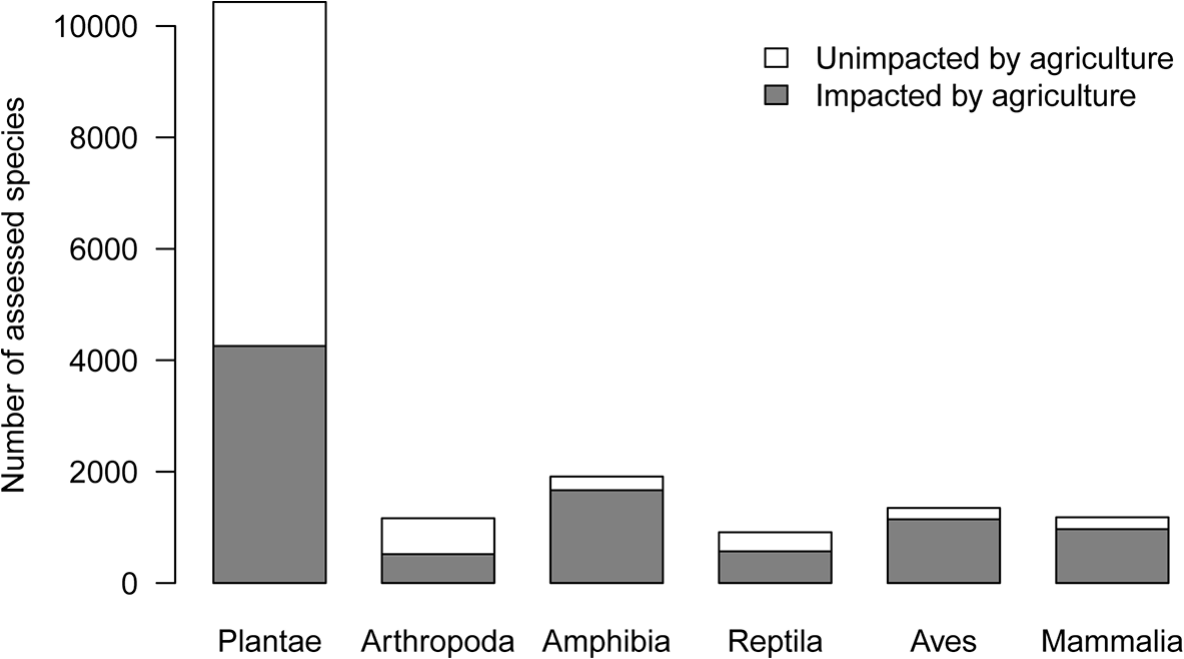
Estimated numbers of threatened species negatively impacted by agriculture. We counted the total number of species classified as either critically endangered, endangered, or vulnerable by the IUCN Red List in different taxonomic units and the number that were assessed to be threatened by at least one agricultural activity [11]. Agricultural activities were annual and perennial nontimber crops, wood and pulp plantations, livestock farming and ranching, logging and wood harvesting, abstracting of surface water (agricultural use), abstraction of ground water (agricultural use), and agricultural and forestry effluents. Many other species may become threatened with agricultural expansion (S2 Fig).

Agriculture’s demand for land drives conversion of natural habitats, and this is arguably its largest environmental cost. Approximately 40% of ice-free land (4,300 million ha) is already covered by crops or used to raise livestock [7], and an additional 2.7–4.9 million ha of cropland annually, the size of a small European nation, may be required to feed the world’s population by 2030 [1,9]. Conversion of land for agriculture is estimated to account for 80% of global deforestation [10], and ca. 53% of terrestrial species assessed as threatened by the International Union for Conservation of Nature (IUCN) [11] are negatively impacted by agriculture (Fig 2). Land conversion also reduces the size of the terrestrial carbon sink. Global simulation models predict that 24% and 10% less carbon is stored in vegetation and soil, respectively, than if present-day landscapes retained their natural vegetation [12].

Agriculture also contributes more to other forms of environmental degradation than any other economic sector. Between 30%–35% of global greenhouse gas (GHG) emissions come from agriculture [3], and crop irrigation accounts for 70% of the world’s freshwater withdrawals [13]. The use of synthetic nitrogen fertiliser has increased nearly 21-fold since 1950, and more nitrogen is now added to agricultural soils than from natural processes [14]. Virtually all human-derived nitrogen is lost to the atmosphere or receiving waters [14,15]. This eutrophication suffocates aquatic ecosystems [16]. Phosphorus from fertiliser and livestock manure [14,17], pesticides [18–20], and nanoparticles [21,22] are also exacerbating environmental pollution. Additionally, poor agricultural management causes soil degradation, reducing agricultural productivity and creating further demand for nutrients, water, and land conversion [23,24].

Solutions for limiting agriculture’s environmental costs are increasingly well understood. These include reducing land clearance by improving yields of existing crops, minimizing excess nutrient and water use, and shifting production from livestock towards crops [3]. Implementing these practices for only a handful of commodities in a subset of the world can have a large environmental benefit [25]. The success of these practices, however, depends on how well policy puts them into practice.

## Challenges to Limiting Agriculture’s Environmental Costs

Designing and implementing policies to reduce agriculture’s environmental costs is difficult. Like other industries, farmers and the businesses reliant upon them, such as input suppliers, processors, and retailers, have a rational self-interest in maximising economic returns and oppose policies that threaten their viability [26,27]. In developed economies, this has historically allowed them to organise into focused lobby groups with considerable advantage over diffuse public interests seeking to safeguard the environment [28,29]. Governments rarely benefit from challenging agri-food lobbies over environmental issues that lack immediate returns for the public good. These lobbies reward government for less demanding environmental policies [30,31], and tailoring policies appropriate for the range of physical and social conditions that farms occupy is costly [26,27]. Concerns about foodborne illness (including genetically modified organisms), environmental impacts (e.g., pesticide use), and animal welfare have ceded some power in the policy arena of developed countries from producers to consumers [32], and increasingly to large retailers, but existing governance structures remain highly susceptible to farmer interests despite spatiotemporal variation in power relations [29]. For example, corporate lobbying efforts in the agricultural sector were deemed effective by 52% of respondents in a 2013 survey of nearly 600 European Union (EU) decision makers, and >80% effectiveness was reported in Denmark, Latvia, and the United Kingdom [33]. Policymakers therefore face major challenges in aligning the private interests of the agri-food sector with public interest in environmental goods across the diversity and extent of farm entities.

In addition to political challenges, measures that do succeed in reducing the environmental harms of agriculture in one place can perversely increase them elsewhere. This is because, in a global market, local policies or technologies that cause agricultural production to carry a greater opportunity cost than using land for environmental goods will displace production to regions where these costs are lower [34]. For example, regulations limiting fertilisers can reduce local water pollution and GHG emissions. But because such measures may reduce yields (i.e., tonnage of production per unit area), food demand will be met elsewhere, leading to intensification and land clearance that potentially creates a net environmental loss [35]. Incentivising low-intensity systems, seen as beneficial to biodiversity conservation in regions such as Europe, may similarly limit production and displace environmental impacts while also lacking the full benefits of natural ecosystems [36]. Efforts to limit the environmental costs of agriculture must therefore consider the environmental impacts beyond farm boundaries as well as the consequences for food production.

## Potential Resolutions to the Agriculture–Nature Conflict

Agricultural policy with explicit environmental objectives is generally organized around three approaches (Table 1 and expanded upon in S1 Table; see Box 1 for an exception):

1. regulations, such as limits on pesticide use or abstracting water, which can be enforced through penalties and conditions placed on financial support to farmers;
2. community-based approaches, which support farmers and local stakeholders to work collectively in addressing environmental impacts; and
3. economic instruments, which pay farmers directly or create markets for adopting practices that minimise environmental impacts and provide noncommodity outputs beyond those required by existing regulation; tariffs can also be used to internalise environmental costs.

**Table 1:** Successes associated with different policy approaches and the local^1^ obstacles to their implementation.

Approach	Success story	Obstacles
*Regulations*	Critical Counties programme launched in 2008 by Brazilian government suspended access to credit for farmers in counties with the highest deforestation rates. This stimulated collective actions to conserve native vegetation, resulting in deforestation rates dropping to <20% of previous 10-yr average [40].	Complexity, enforceability, cost of implementation. Where compliance is linked to payments, as in EU’s Common Agricultural Policy, clear environmental standards and operational guidance required at farm level [41]. Payments must also incorporate regional and/or sectorial variation in the cost of compliance.
*Community-based*	Landcare Australia is among the best known partnerships between communities, government, and organisations. Established by a $360 million programme in 1989 and engaging about 30% of farming community at its height [42]. Notable successes improving water quality, reducing soil degradation, restoring habitat [43].	Delivering social and/or short-term economic benefits to individual farmers; lack of these benefits has limited uptake of Landcare practices in Australia [44,45].
*Economic instruments*	Conservation Reserve Program (CRP) established in 1985 by US government provides farmers with annual rental payments for removing land most sensitive to agricultural impacts from production for ≥10 years. Currently, >10 million ha enrolled at annual cost of ca. US$2 billion [46]. CRP has eliminated millions of tonnes of GHG emissions and fertiliser additions and established new habitat supporting tens of millions of birds and other wildlife [47]. Gains arisen despite conversion of non-cropland into production predicted over area equal to 20% of CRP land [48].	Can perform poorly if farmers paid for actions rather than actual environmental outcomes associated with their land [49]. For example, temporary retirement schemes emerged initially in EU to reduce overproduction. As no environmental outcome was explicitly targeted, farmers tended to retire least profitable land that was often the poorest quality, limiting environmental benefits [50,51].

^1^Globally, the major obstacle for all approaches is to avoid displacing production and causing environmental degradation elsewhere.

**Box 1. Abandoning the Agricultural Sector to Market Forces**

New Zealand radically altered its agricultural policy in 1984 to embrace minimal government intervention. It now relies exclusively on regulation to minimise the environmental impacts of agriculture while providing among the least support to domestic producers out of all developed countries (ca. 0.5% of the value of production; S1 Text). Consequently, the natural environment has been left vulnerable to the pressures of market forces, which are rapidly accelerating production and land conversion with increasing demand for dairy products in particular. The rate of conversion of indigenous grassland to exotic pasture in the South Island has increased by 67% from the period between 1990–2001 to 2001–2008 [52], and New Zealand leads the world in livestock emissions per capita, exceeding the global total across developed countries by more than 13-fold [53].

The nonprescriptive Resource Management Act (RMA) is the primary national legislation intended to oversee natural and physical resources, with land regulation devolved to elected district councils. Although the RMA tasks councils with “maintaining” biodiversity in balance with resource development, they generally expedite the latter. Fewer than 5% of agricultural sector consents requiring biodiversity or ecosystem services maintenance were recently found to comply with conditions [54]. Limited resources are often blamed for poor enforcement, but political interference also occurs [55]. Other regulations, such as the Wildlife Act and Native Plant Protection Act, are rarely invoked, and the latter does not apply to landowners [55].

New Zealand also has a dedicated judicial system to contest decisions made under the RMA. While this system has delivered several notable “wins” for the environment (e.g., [56]), many local actions escape scrutiny. In practice, biodiversity protection by rural landowners occurs mainly on small, voluntary, set-asides of residual unproductive land (known as covenants) and through patchy predator-trapping and stock fencing activities, supported by limited local and national funds. It appears that the laissez-faire approach to the agricultural sector does little to safeguard the environment.

Farmers will be motivated to accept one or more of these approaches where they incur small socioeconomic costs relative to the benefits that they receive from achieving environmental objectives [37,38]. Eliminating financial support for farmers to maximise agricultural production is important in helping to reduce the socioeconomic costs of adopting environmental practices. Reforms in developed economies since the 1980s are now supporting producer incomes more through direct payments than by subsidising production or protecting domestic prices [2,39] (Box 2).

Even with farmer acceptance, success of any policy approach will ultimately depend on whether farmers maintain or boost yields on existing agricultural lands rather than convert new natural lands to production. Halting local land conversion can be achieved with strict enforced regulations (Table 1), but in a globalized market, changes in the local supply of a given cash crop will lead to changes in demand and supply in other distant locations [59]. Trends in cereal production in 19 countries between 1990 and 2005 clearly show this link between local actions and global consequences. These countries managed to boost cereal yields on existing lands while removing cropland from production, but without fully meeting local demand. Per capita cereal imports consequently increased by 12-fold in these countries compared with those lacking land retirement policies [60], clearly suggesting that production was displaced elsewhere. Where subsistence agriculture is important, limiting its local production may similarly boost reliance on cash crops produced abroad [61]. National governments implementing any of the three main policy approaches need to begin working in a coordinated way to combat land use displacement.

**Box 2. Environmental Harms of Agricultural Price Support**

Price support policies, such as import tariffs or minimum commodity prices, encourage overproduction in the absence of supply management. This strains natural resources and makes marginal land artificially profitable [57]. In the US, the federal government historically managed the dairy industry by guaranteeing to purchase milk if its market price fell below a set value. Modelling revealed that this would drive abandonment and reversion to natural forest across thousands of hectares of land in the state of Wisconsin if milk prices were decreased by only 5%–15%, with millions saved in water treatment and government expenditure [58]. The US has now adopted a strategy of paying producers should their margins fall beneath national averages, likely with similar environmental consequences.

### Finding the Right Mixture of Solutions

Maintaining the status quo with agricultural policy is unsustainable for the environment [3,35,62]. On their own, regulations and community approaches will offer little help if damaging the environment remains financially lucrative because the associated penalties are trivial, governments continue to subsidise production (Fig 1, S1 Fig), and farmers are not accountable for the environmental costs of their actions [63–65] (Box 3). Economic instruments, such as payments for public goods, are also contentious, especially for biodiversity conservation [66–68]. Positive outcomes often arise only from certain actions, such as enhancing specific food resources or nesting habitat [69], and catch-all interventions may avoid targeting rare species [70,71]. Removing production subsidies and making farmers financially responsible for their environmental impacts, such as with markets for public goods, can help local outcomes but must avoid curbing intensification because this will encourage land conversion elsewhere to meet demand for food [35].

**Box 3. Balancing Large-Scale Incentives for the Rural Poor with Strict Penalties for Agri-business**

China—the world’s largest agricultural producer—offers a stark contrast in government policy to that implemented by New Zealand described in Box 2. The country’s economy is heavily controlled by centralised government and relatively free from the political influence wielded by farmers lobbies. Government can therefore act largely in its own self-interest, which may align more often with the public good. This has allowed the Chinese government to implement environmental policies on scales that would be virtually impossible elsewhere in the world. The Grain to Green Project is one example, having converted nearly 9.1 million hectares of cropland into forest whilst incentivising farmers to participate with grain and cash subsidies [72]. This permanent land retirement scheme has reduced soil erosion and increased carbon sequestration at landscape scales [73]. China is also adopting a stricter approach to large industrial agriculture. In 2014, the country’s top legislature revised the national environmental protection law for the first time in 25 years. The revisions included harsher penalties to deter companies from accepting fines rather than investing in pollution control [74]. Whether enforcement follow suits remains to be seen.

Ruhl [26] argued that successful agricultural policies will target large industrial operations that can be readily regulated while incentivising changes in production practices by small farmers without compromising their livelihoods. Recent developments in China are therefore promising, but the country’s agricultural sector still causes major environmental harm, including poor water quality in almost all rivers and lakes [75], and nearly 40% more GHG emissions per tonne of nitrogen fertiliser than in Europe [76]. Subsidies on inputs such as fertilisers and pesticides are largely to blame as they promote overuse. Fertilisers are estimated to be overused by 30%–60% [77], yet nearly CMY¥108,000 million (US$17,000 million) was spent in 2012 on just one scheme to help farmers purchase more of these inputs [78]. Removing harmful financial subsidies would help greatly in aligning the country’s agricultural and environmental aims.

We assert that conflict between agriculture and the environment will be best resolved by policies dedicating high-quality habitat towards nature conservation, while encouraging intensive production on existing farmland with stringent limits on environmental impacts [35,36,79–81]. Measures that make farmland itself more benign—so called “land-sharing” approaches—also deliver local environmental and social benefits [82,83], but can reduce agricultural yields and cause land conversion elsewhere, effectively displacing environmental burdens [80]. Intensifying production on existing farmland may thus be a better option because it avoids displacing impacts and can allow other land to be freed for nature conservation and restoration, with no net loss in agricultural production.

Our consideration of policy instruments approaches and the obstacles to their success (Table 1) suggests that our proposed resolution would practically entail a combination of punitive measures, such as taxes that reduce existing overuse of farm inputs [84], with positive incentives linked to measurable outcomes [49], such as for permanently removing land from production. Taxes on farm inputs can improve upon existing payments to limit input use because they do not explicitly cap nutrient, pesticide, and water use. Without input limits, some farmers may find it profitable to intensify existing production, despite the added taxes, while others might profit more from reducing resource consumption. Across the landscape, yields will be maintained, and potentially increased without additional land conversion. For example, water surcharges in Mexico are used to pay landowners to retain forested watersheds. Displaced land conversion from exploitation of communal properties and changing market pressures reduced the environmental benefits of this programme by only ca. 4% [85]. By enabling farms to respond differently to the same policy, input taxes carry an additional advantage over traditional regulations that hold all farmers to the same standard and ignore sectorial diversity [86]. An aligned but more audacious approach would be to replace taxes on income with taxes on environmental consumption. The idea is to shift from taxing the production of private wealth toward taxing the consumption of public environmental goods, thus promoting economic growth from sustainable use rather than depletion of natural capital. Brown et al. [55] have proposed that such a system would most heavily tax intensive land uses that generate the most negative environmental impacts, while areas of intact ecological function would entitle the owners to a rebate. This approach might encourage farmers to work intensively with the most productive land but set aside and restore less productive areas with net gains for the environment.

**Fig 3:**
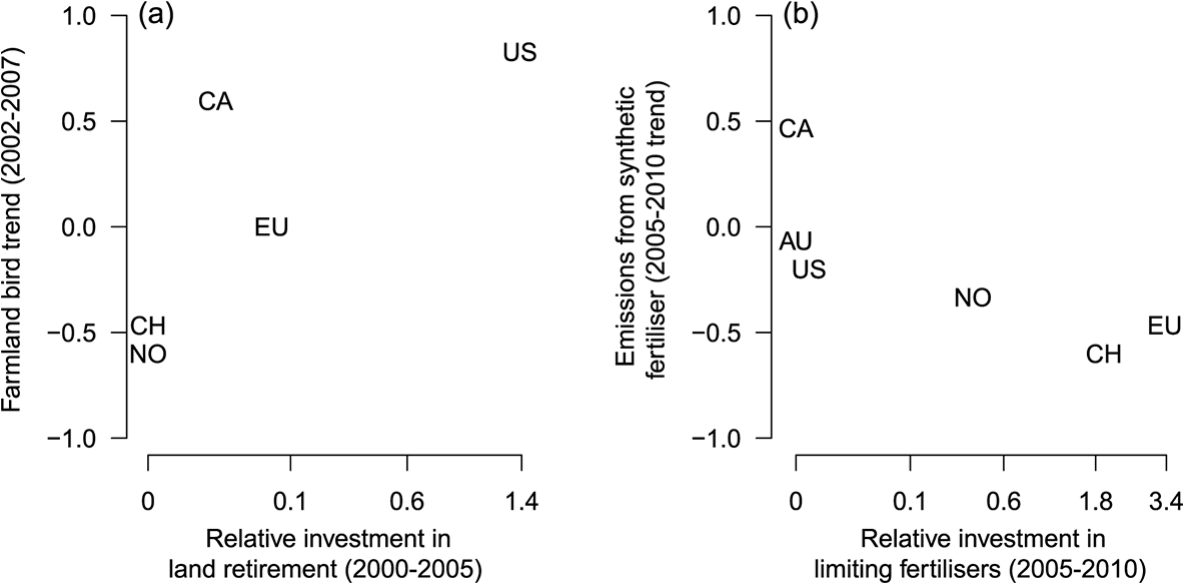
Improvements in environmental performance of agriculture correlate with investments in agri-environment schemes (AES). Temporal trend in (**A**) farmland bird populations and (**B**) GHG emissions from synthetic fertiliser were calculated over time for each country using Kendall’s *τ* (see S1 Text for full methods). Investments in AES were scaled by the ratio of investments in AES relative to financial transfers to agricultural producers for all other purposes so as to account for the fact that environmental improvements can be offset by policies that maximise production. Values were then summed across 5-y periods with a pre-emptive 2-y lag for land retirement. Habitat quality will take at least one growing season to improve following land retirement and any changes in bird populations from improved breeding success will thus largely be measurable the following year, i.e., two years later [87,88]. Spearman’s rank correlation: *ρ* = 0.95, *p* = 0.005 and *ρ* = -0.93, *p* = 0.008 for (**A**) and (**B**), respectively. Points are responses in individual countries: AU = Australia [data available for (**B**) only], CA = Canada, EU (aggregated together), NO = Norway, CH = Switzerland, and US. Data used to make this figure are provided in S1 Data.

Evidence at national levels suggests that investments in land retirement and input-limiting schemes, consistent with our suggested policy approach, do deliver local environmental benefits. To illustrate this, we examined trends in two environmental responses, farmland bird populations and GHG emissions from synthetic fertilisers, across countries with similar agricultural patterns, i.e., dominated by livestock and arable crops (see S1 Text for methods). We calculated the annual, per hectare monetary investment in a specific environmental outcome beyond that required by regulation from a detailed inventory of government policies compiled by the OECD (annotated in S1 Data). As expected, because habitat loss is a major threat to farmland and grassland biota, we found that farmland birds benefited as more money was invested in retiring agricultural land (Fig 2). We also found that total emissions of N_2_O declined with increasing investment in reducing fertiliser use (Fig 3). While cursory in nature, these results suggest that targeted policies have the potential to deliver positive environmental outcomes.

Measures delivering environmental benefits must also be coupled with mechanisms that promote intensification to avoid displacing production elsewhere, and they must do so with fewer external costs. Research suggests increased yields and better environmental practices are possible by improving nutrient and water management and breeding more efficient crops [3]. Reductions in food waste and human diets with less livestock and dairy consumption can also help meet rising food demands without increasing resource use [89,90]. Dietary change is, however, contentious. Governments may need to consider policies that tax livestock products and/or subsidize the development of livestock-free protein sources, e.g., [91], in order to dislodge entrenched norms.

Here, we have laid out a vision for limiting the environmental impacts of agriculture, yet at least two major challenges remain. First, winning political support for policy change remains a fundamental requirement to any of our proposed reforms. Ultimately, democratically elected governments, while not immune to the lobbying power of agribusiness, will generally respond to electoral pressures [31]. Electorates may express little desire for policy change if they are poorly informed, unmotivated, or misled by industry about limiting the environmental costs of agriculture. Second, implementation of our policy mixture will vary spatially. For example, successful land retirement depends on features of the surrounding landscape, such as habitat connectivity [92], and can reduce yields in regions where the most productive land also has the most valuable environmental assets. Nonetheless, improvements in the management of land resources in developing countries [93], new technologies that optimally allocate farm inputs to reduce overuse [94], advances in crop breeding [95], and tools for better conservation planning [96] give us hope that innovations exist for meeting the need for more sustainable and intensive agriculture.

## Supporting Information

S1 Data Investments in different agricultural policies used to generate Figs 1 and 3. (XLSX)

S1 Fig Proportion by which government policies increase gross returns to farmers as compared with the absence of government intervention. Blue segments show the nominal rate of assistance (NRA) estimated in 2011 for 137 countries with agricultural sectors comprising >5% of gross domestic product [97]. NRA is equal to the difference in price of agricultural commodities that have been adjusted for changes in value associated with domestic policies, such as import tariffs or production subsidies, and the global undistorted price at the country border and expressed as fraction of the undistorted price [98]. It provides data on many more countries than the OECD producer support estimate and is also advantageous as it is expressed relative to the undistorted rather than distorted value [98]. Countries with NRA equal to zero (entirely white) provide no support to their agricultural sector, have a negative NRA (i.e., taxation), or are low to middle income (gross national income per capita of <$4,085 USD). Sizes of symbols are scaled to the total area under agricultural production.
(DOCX)

S2 Fig Estimated numbers of near-threatened species negatively impacted by agriculture. We counted the total number of species classified as either near-threatened or conservation-dependent in different taxonomic units and the number that were assessed to be threatened by at least one agricultural activity [11]. Agricultural activities were annual and perennial nontimber crops, wood and pulp plantations, livestock farming and ranching, logging and wood harvesting, abstracting of surface water (agricultural use), abstraction of ground water (agricultural use), and agricultural and forestry effluents.
(DOCX)

S3 Fig Correspondence between the OECD Producer Support Estimate (PSE) database and WTO notifications. Points are country-level estimates of: (A) support for AES; (B) total producer support (PS); and (C) value of production (VOP). Estimates are reported in a mixture of local currencies and US dollars, depending on country. *n* = 12 countries, except for (B), where the Ukraine is omitted because PS is negative due to the way in which market price support is calculated (see S1 Text).
(DOCX)

S1 Text Supporting Methods. (DOCX)

S1 Table Pros and cons of measures used to mitigate conflict between agriculture and the environment. We also predict the most likely consequences of these measures for food production (Δ yields), recognizing that some measures can vary substantially, such as if they produce environmental goods that promote yields (e.g., pollinators) and require compliance with conditions that also reduce yields (e.g., pesticide bans). Examples of where these measures are used are not intended to be exhaustive.
(DOCX)

## Abbreviations

AES: agri-environment schemes
CRP: Conservation Reserve Program
GHG: greenhouse gas
IUCN: International Union for Conservation of Nature
NRA: nominal rate of assistance
OECD: Organisation for Economic Cooperation and Development
PS: producer support
PSE: producer support estimate
RMA: resource management act
VOP: value of production
WTO: World Trade Organization
