# Supplementary material for: Resolving Conflicts between Agriculture and the Natural Environment
Source: PLoS Biol. 2015 Sep 9;13(9):e1002242. doi: 10.1371/journal.pbio.1002242 (PMC4564228; doi:10.1371/journal.pbio.1002242)
Supplement: S1 Fig — Blue segments show the nominal rate of assistance (NRA) estimated in 2011 for 137 countries with agricultural sectors comprising >5% of gross domestic product [97]. NRA is equal to the difference in price of agricultural commodities that have been adjusted for changes in value associated with domestic policies, such as import tariffs or production subsidies, and the global undistorted price at the country border and expressed as fraction of the undistorted price [98]. It provides data on many more countries than the OECD producer support estimate and is also advantageous as it is expressed relative to the undistorted rather than distorted value [98]. Countries with NRA equal to zero (entirely white) provide no support to their agricultural sector, have a negative NRA (i.e., taxation), or are low to middle income (gross national income per capita of <$4,085 USD). Sizes of symbols are scaled to the total area under agricultural production. (DOCX) [file pbio.1002242.s002.docx]

**Supporting Information for ‘Resolving Conflicts between Agriculture and the Natural Environment’**

Andrew J. Tanentzap, Anthony Lamb, Susan Walker, Andrew Farmer

**
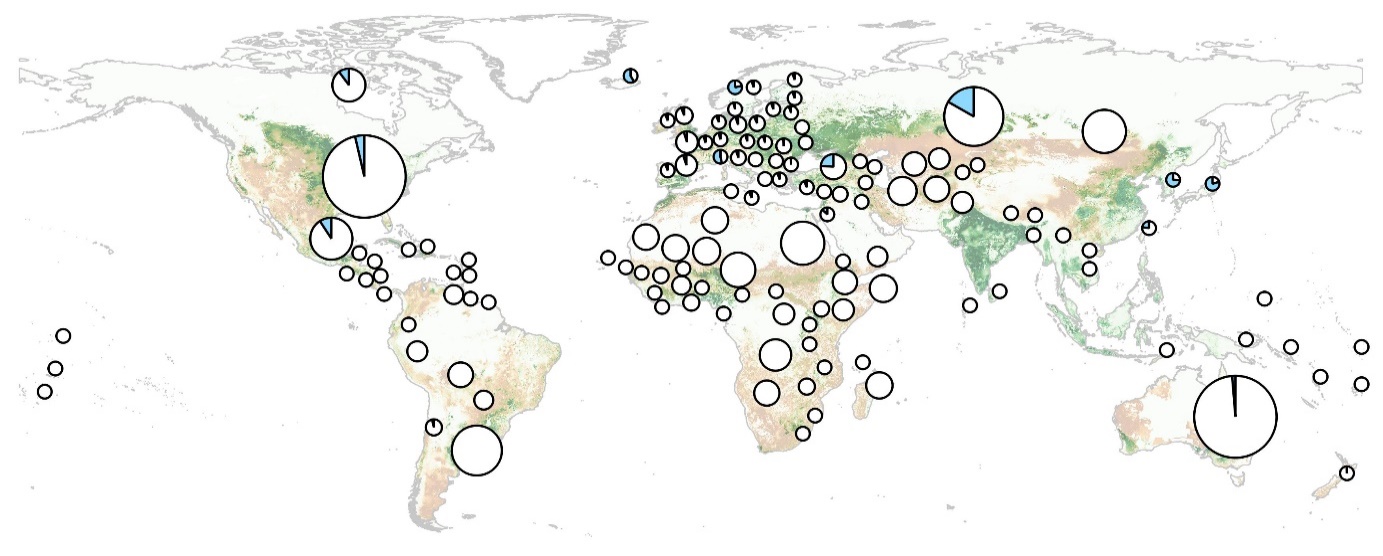
**
