# Supplementary material for: Resolving Conflicts between Agriculture and the Natural Environment
Source: PLoS Biol. 2015 Sep 9;13(9):e1002242. doi: 10.1371/journal.pbio.1002242 (PMC4564228; doi:10.1371/journal.pbio.1002242)
Supplement: S2 Fig — We counted the total number of species classified as either near-threatened or conservation-dependent in different taxonomic units and the number that were assessed to be threatened by at least one agricultural activity [11]. Agricultural activities were annual and perennial nontimber crops, wood and pulp plantations, livestock farming and ranching, logging and wood harvesting, abstracting of surface water (agricultural use), abstraction of ground water (agricultural use), and agricultural and forestry effluents. (DOCX) [file pbio.1002242.s003.docx]

**Supporting Information for ‘Resolving Conflicts between Agriculture and the Natural Environment’**

Andrew J. Tanentzap, Anthony Lamb, Susan Walker, Andrew Farmer

**S3 Fig. Correspondence between the OECD PSE database and WTO notifications.** Points are country-level estimates of: (A) support for agri-environmental schemes (AES); (B) total producer support (PS); and (C) value of production (VOP). Estimates are reported in a mixture of local currencies and US dollars, depending on country. *n* = 12 countries, except for (B), where the Ukraine is omitted because PS is negative due to the way in which market price support is calculated (see Text S1).
