# Supplementary material for: Resolving Conflicts between Agriculture and the Natural Environment
Source: PLoS Biol. 2015 Sep 9;13(9):e1002242. doi: 10.1371/journal.pbio.1002242 (PMC4564228; doi:10.1371/journal.pbio.1002242)
Supplement: S3 Fig — Points are country-level estimates of: (A) support for AES; (B) total producer support (PS); and (C) value of production (VOP). Estimates are reported in a mixture of local currencies and US dollars, depending on country. n = 12 countries, except for (B), where the Ukraine is omitted because PS is negative due to the way in which market price support is calculated (see S1 Text). (DOCX) [file pbio.1002242.s004.docx]

**Supporting Information for ‘Resolving Conflicts between Agriculture and the Natural Environment’**

Andrew J. Tanentzap, Anthony Lamb, Susan Walker, Andrew Farmer
