# Supplementary material for: Resolving Conflicts between Agriculture and the Natural Environment
Source: PLoS Biol. 2015 Sep 9;13(9):e1002242. doi: 10.1371/journal.pbio.1002242 (PMC4564228; doi:10.1371/journal.pbio.1002242)
Supplement: S1 Text — (DOCX) [file pbio.1002242.s005.docx]

**Supporting Information for ‘Resolving Conflicts between Agriculture and the Natural Environment’**

Andrew J. Tanentzap, Anthony Lamb, Susan Walker, Andrew Farmer

**S1 Text. Supporting Methods.**

*WTO-OECD harmonized agri-environment database*

We generated a single database tracking financial support for agri-environmental schemes, i.e. practices that farmers undertake to achieve specific environmental objectives beyond those required by regulation [1]. This drew upon two main databases of global support to agricultural producers. The first was the Agricultural Information Management System (http://agims.wto.org/) managed by the World Trade Organization (WTO). Under the Uruguay Round Agreement on Agriculture, an international treaty that aims to limit trade-distorting agricultural policies, government support of domestic agricultural producers must be annually disclosed to the WTO Committee on Agriculture. Policies are classified as those that minimally distort production (“green box” payments, such as environmental programmes), involve production limits (“blue box”, such as production-limiting programmes for price protection), support development (“development box”, only applicable for developing countries), or distort production (“amber box”, such as direct payments for yield). All the policies in the ‘amber’ box are then summed to produce an aggregate measurement of support (AMS). The second database that we utilized was the Organisation for Economic Co-operation and Development (OECD) Producer and Consumer Support Estimates database (http://www.oecd.org/tad/agricultural-policies/producerandconsumersupportestimatesdatabase.htm). The OECD database tracks a smaller set of countries for which policies providing economic support to agricultural producers are annually compiled. Data are contributed by member countries, with OECD experts collating additional information from budgetary and government documents where necessary. OECD staff classify support payments based on their implementation criteria, such as if they are based on input use, area/animal numbers, non-commodity criteria, etc., and use these to derive a producer support estimate (PSE), which is the total sum of transfers to agricultural producers from consumers and taxpayers.

There are important differences between the WTO and OECD systems that can make them difficult to compare. First, the two systems employ different methods to estimate market price support (MPS). The OECD compares the gap between current domestic and reference prices for all policies that alter domestic prices, e.g. trade protection, taxation, and commodity interventions. By contrast, the WTO only estimates MPS if domestic prices are directly administered and there does not need to be any association between administrated prices and other policies, such as trade protection [2]. This can result in substantial differences when MPS is incorporated into calculations of PSE and AMS [3,4]. Second, differences in data classification and sources help explain why WTO notifications are generally but not always consistent with OECD estimates of producer support [2,3]. The OECD provide much clearer information on the purpose of payments than the WTO system, which is only interested in the outcomes of policies on international trade. Agri-environmental programmes may also be used in WTO notifications to disguise protection rather than deliver environmental benefits [5], especially as each country reports very different amounts of information about domestic policies and these are not standardized as in the OECD. Finally, OECD data are generally updated more often than the WTO, because notifications submitted to the WTO are published only as they are submitted by member governments and so can suffer from substantial delays. By contrast, OECD data are derived by independent experts that synthesize different data than that necessarily included in the WTO notifications reported by member governments. While all these reasons would suggest that the OECD estimates are superior to those derived from the WTO for the purposes of our study, we used the latter in our study because it included additional countries.

Despite estimating total domestic support, neither the WTO nor OECD estimate support for agricultural producers to undertake practices that achieve specific environmental objectives extending beyond those required by regulation, i.e. agri-environmental schemes (AES). Therefore, we classified all policies reported in the two systems as either AES or “other”. First, we analysed all of the most recent domestic support notifications for WTO members, selecting those that disclosed the total value of agricultural production (VOP) or VOP for primary commodities in the case of Argentina and India. This resulted in data for 23 countries and the EU, accounting for 73% of global production in 2013 [6], and ranging from some of the largest to smallest agricultural producers. For each notification, we reviewed all programmes transferring funds to domestic producers and summed the value of those that could be classified as AES based on either descriptions in the WTO notifications or supporting documents from websites of the respective governments. Total support to domestic producers was calculated as the reported AMS contribution plus contributions towards all other types of producer support mentioned in the “green box”, “blue box” and “development box”. To ensure that this estimate of producer support was comparable to the OECD PSE, we only summed those policies that we believed to represent transfers to individual producers rather than the entire agricultural community, which the OECD classifies as part of its General Services Support Estimate. For the OECD databases, we downloaded country-level files and used supporting documents describing different policies to identify those that could be classified as AES; five countries already had classified until 2008 [1]. We also extracted the total amount of producer support (i.e. PSE) and VOP at the farm gate. In total, we had data for 20 countries and the EU, accounting for 65% of global production in 2013 [6]. We retained data only for the most recent year reported, which ranged from 2008 to 2013, and standardized all monetary values to US dollars using the mean of bid/ask conversion rates reported for the year of interest by Oanda (www.oanda.com). All our policy classifications and local currency conversions are provided in S1 Data.

Economic estimates were very similar for the 12 countries found in both the WTO and OECD datasets (S3 Fig.). First, contributions towards AES were closely correlated, showing that our classification of WTO notifications generally matched work by the OECD (*r* = 0.95, *p* < 0.001). There were however deviations for a subset of countries (e.g. China, Korea, Japan, Canada), with the WTO data generally overestimating contributions towards AES (panel A in S3 Fig). This likely arose from the fact that programmes were consolidated into groups in the WTO notifications from which it was difficult to separate AES from other measures of producer support, but these information were available to the OECD. Second, we similarly found close correspondence between the OECD PSE and our estimated measure of producer support from the WTO data (*r* = 0.97, *p* < 0.001; panel B in S3 Fig.). Any differences that arose were because of variation in the way that MPS was calculated. For example, PSE was negative in the OECD database for the Ukraine in 2011 but not in the WTO data, which does consider export restrictions that have restricted the potential for producers to fully benefit from external markets [7]. Finally, we found the strongest correlation between the OECD and WTO databases for VOP (*r* ~ 1.00, *p* < 0.001; panel C in S3 Fig.). Slight discrepancies arose between the estimates because the WTO notifications sometimes report figures on a different calendar than the OECD, e.g. calendar or marketing year versus financial year.

*Farmland bird indices*

We calculated temporal changes in the farmland bird index (FBI) of different countries for a period immediately following the implementation of different AES. Farmland bird indices are derived by aggregating assessments of population trends across multiple species that use farmland for nesting or breeding [8]. Values are typically arbitrary units standardised to some baseline measurement, such as a FBI = 100 in 2000. This enables comparison in the trends among countries. In Europe, the FBI is comprised from 37 species that are monitored and have their trends aggregated across countries with relevant survey data in that year [9]. In Canada and the US, 28 and 24 grassland breeding birds are monitored, respectively [10,11], and data are provided by the OECD [12].

*Synthetic fertiliser emissions*

We collated data on total emissions of N_2_O from synthetic nitrogen fertilizer added to agricultural soils from the FAO [5]. This included N_2_O released from nitrification and de-nitrification by microbial processes at the site of fertiliser addition and after volatilization and leaching. Measurements of annual synthetic fertiliser applications are multiplied by standard emissions rates for unit of fertiliser used by the IPCC [13]. A similar series of standard equations is use to convert fertiliser applications into the amount that volatilises and is lost through runoff and leaching [13].

*Trend detection*

We described monotonic change in the environmental time series of each country using the Mann-Kendall *τ* statistic. This is a widely used non-parametric statistic [14], with values ranging from -1 to 1, indicating strong negative and positive trends respectively. We then used these *τ* statistics – one for each country – to test the hypothesis that sustained investment in AES would be correlated with environmental responses.

Our focus was on testing the explicit hypotheses that farmland birds would benefit from removing land from production, because habitat loss is the greatest threat to farmland and grassland biota (Fig. 2), and that greenhouse gas emissions would be reduced by limiting farm inputs. For this reason, we did not relate our environmental responses to other types of AES, though our dataset does permit these analyses (S1 Data). For the farmland birds, we expected based on other studies that there would be a time lag before land retirement would improve habitat quality [15,16], and so calculated trends over a period that was shifted forward 2 years from investments in AES.

We did not directly relate FBI or fertiliser emissions of a given year to the relative investment in AES in that year because the state of the environmental indicators depends on their levels prior to the AES being implemented. For example, consider a FBI = 96 at *t* = 1. FBI could have been 85 prior to AES investment at *t* = 0, in which case the FBI responded positively, or FBI could have been 106 at *t* = 0, in which case the FBI responded negatively. The *τ* statistic avoids this by considering the overall direction of change during the period of AES investment. This statistic is also less sensitive to outlier years than mean annual change in a given indicator; although the two were closely correlated for FBI and fertiliser emissions: *ρ* = 0.90, *p* = 0.042 and *ρ* = 0.83, *p* = 0.029, respectively, where *p* is from a one-tailed test.

*References*

1. Vojtech V (2010) Policy measures addressing agri-environmental issues. Paris: Organisation for Economic Co-operation and Development. 42 p.
2. Mittenzwei K, Josling T (2012) Comparative analysis of data frameworks for agricultural policy analysis. Oslo: Norwegian Agricultural Economics Research Institute. 82 p.
3. Diakosavvas D (2002) How to measure the level of agricultural support: comparison of the methodologies applied by OECD and WTO. In: Legg W, Li X, Patterson M, Taylor S, Trzeciak-Duval A, editors. Agricultural policies in China after WTO accession, Paris: Organisation for Economic Co-operation and Development. pp. 217-245.
4. Effland A (2011) Classifying and measuring agricultural support: identifying differences between the WTO and OECD systems. Washington, DC: US Department of Agriculture. 24 p.
5. Diakosavvas D (2003) The greening of the WTO green box: a quantitative appraisal of agri-environmental policies in OECD countries. Available: http://www.ecostat.unical.it/2003agtradeconf/Contributed%20papers/Diakosavvas.PDF via the Internet. Accessed 2015 March 14.
6. Food and Agriculture Organization of the United Nations (2014) Database: FAOSTAT. Available: http://faostat3.fao.org via the Internet. Accessed 2015 Mar 14.
7. Kwieciński A, Brooks J, Cervantes-Godoy D, Jones D, Melyukhina O, et al. (2009) Agricultural policies in emerging economies 2009. Paris: OECD. 192 p.
8. Gregory RD, van Strien A, Vorisek P, Meyling AWG, Noble DG, et al. (2005) Developing indicators for European birds. Philos Trans R Soc B 360: 269-288.
9. Statistical Office of the European Commission (2014) Database: Eurostat. Available: http://ec.europa.eu/eurostat/data/ via the Internet. Accessed 2015 Mar 14.
10. North American Bird Conservation Initiative Canada. (2012) The state of Canada’s birds, 2012. Ottawa: Environment Canada. 36 p.
11. North American Bird Conservation Initiative (2014) The state of the birds 2014 report. Washington, DC: US Department of Interior. 16 p.
12. OECD (2013) OECD compendium of agri-environmental indicators. Paris: OECD. 190 p.
13. Intergovernmental Panel on Climate Change [IPCC] (2006) 2006 IPCC Guidelines for National Greenhouse Gas Inventories. Hayama: Institute for Global Environmental Strategies.
14. Yue S, Pilon P, Cavadias G (2002) Power of the Mann–Kendall and Spearman’s rho tests for detecting monotonic trends in hydrological series. J Hydrol 259: 254-271.
15. Chamberlain DE, Fuller RJ, Bunce RGH, Duckworth JC, Shrubb M (2000) Changes in the abundance of farmland birds in relation to the timing of agricultural intensification in England and Wales. J Appl Ecol 37: 771-788.
16. Tscharntke T, Batáry P, Dormann CF (2011) Set-aside management: How do succession, sowing patterns and landscape context affect biodiversity? Agric Ecosyst Environ 143: 37-44.
