# Supplementary material for: Resolving Conflicts between Agriculture and the Natural Environment
Source: PLoS Biol. 2015 Sep 9;13(9):e1002242. doi: 10.1371/journal.pbio.1002242 (PMC4564228; doi:10.1371/journal.pbio.1002242)
Supplement: S1 Table — We also predict the most likely consequences of these measures for food production (Δ yields), recognizing that some measures can vary substantially, such as if they produce environmental goods that promote yields (e.g., pollinators) and require compliance with conditions that also reduce yields (e.g., pesticide bans). Examples of where these measures are used are not intended to be exhaustive. (DOCX) [file pbio.1002242.s006.docx]

**Supporting Information for ‘Resolving Conflicts between Agriculture and the Natural Environment’**

Andrew J. Tanentzap, Anthony Lamb, Susan Walker, Andrew Farmer

| Measure | Pros | Cons | Where used | Δ yields |
| --- | --- | --- | --- | --- |
| *Regulations* |  |  |  |  |
| ‘polluter-pays’ legislation (laws imposing penalties for violating specific levels of permissible pollution) | can impose large financial costs to polluters making compliance economically unviable | hard to enforce, cannot apply across diverse sectors | Everywhere | –, ↓ (short term) |
| environmental conditions on production support payments | improves compliance with existing legislation; involves producers who might not actively participate in environmental protection [1]; can help maintain high yields where payments are linked to outputs, thereby avoiding land conversion | hard to derive rules to cover large spatial/socioeconomic extent; apply only to some farmers; farmers reliant on support may not be on sensitive land; offset if high financial incentive for poor environmental management or support too weak | EU, Switzerland | – |
| covenants (binding agreement in land deed to deliver environmental outcomes) | can confer long-term protection | voluntary, difficult to sustain protection where more active management needed [2] | Australia, New Zealand | –, ↓ |
| supply chain intervention (producers in a supply chain adhere to specific environmental conditions) | harnesses agribusiness to exert environmental standards on producers [3]; market-driven | voluntary; monitoring compliance can be hard | Brazil | –, ↓ |
| *Community-based* |  |  |  |  |
| collectives (stewardship groups) | knowledge exchange, build social incentives for environmental protection | financial costs of voluntary action; may require dedicated staff that deliver land management knowledge [4] | Australia, EU, Philippines, South Africa | –, ↑ |
| *Economic instruments* |  |  |  |  |
| agri-environment schemes (practices that achieve specific environmental objectives extending beyond those required by regulation) | pay farmers to do something positive, can be designed to local environmental requirements; can support one-off and ongoing activities | voluntary, so cannot be relied upon to deliver all objectives; costly to implement many options at large scales; risk of displaced production causing environmental degradation elsewhere [5] | EU, US, Norway, China | ↓, –, ↑ |
| land retirement | can target areas where alternative habitat creation most needed; can confer long-term or permanent protection | costly to ensure payments tailored to land quality; exports production to places with potentially lax regulation; limited impact if the retirement is short-term [6,7] | US, EU, China | ↓ |
| input taxes (e.g. fertilisers) | work at large spatial-scales; generate revenue for government | unpopular; transfer costs to consumers | EU | – |
| tradable rights (government sets quotas for inputs or environmental goods, and allow contributions towards this quota to transferred among producers in a market) | incentivize resource conservation or production of environmental goods; responsive to changing market conditions; limit total input use | high transaction costs; can be costly to monitor; difficult to value some environmental goods (e.g. biodiversity) at large spatial scales, though technical advances are encouraging, e.g. [8]. | US, Australia, Chile | – |
| consumption taxes | cost-effective; if taxes concurrently removed on income, will facilitate economic growth and social equity as wages no longer taxed disproportionately more than land; incentivize environmental protection on private land; market created on environmental goods | unpopular; politically risky; indirect effects poorly understood | Namibia, EU [9] | ↓ (short-term) |

*References*

1. Organisation for Economic Co-operation and Development [OECD] (2004) Agriculture and the environment: lessons learned from a decade of OECD work. Paris: OECD. 36 p.
2. Fitzsimons JA, Carr CB (2014) Conservation covenants on private land: issues with measuring and achieving biodiversity outcomes in Australia. Environ Manage 54: 606-616.
3. Gibbs HK, Rausch L, Munger J, Schelly I, Morton DC, et al. (2015) Brazil’s soy moratorium. Science 347: 377-378.
4. Selinske MJ, Coetzee J, Purnell K, Knight AT (2015) Understanding the motivations, satisfaction, and retention of landowners in private land conservation programs. Conserv Lett in press.
5. Wunder S, Engel S, Pagiola S (2008) Taking stock: A comparative analysis of payments for environmental services programs in developed and developing countries. Ecol Econ 65: 834-852.
6. Tscharntke T, Batáry P, Dormann CF (2011) Set-aside management: How do succession, sowing patterns and landscape context affect biodiversity? Agric Ecosyst Environ 143: 37-44.
7. Corbet SA (1995) Insects, plants and succession: advantages of long-term set-aside. Agric Ecosyst Environ 53: 201-217.
8. Bateman IJ, Harwood AR, Mace GM, Watson RT, Abson DJ, et al. (2013) Bringing ecosystem services into economic decision-making: land use in the United Kingdom. Science 341: 45-50.
9. Ekins P (1999) European environmental taxes and charges: recent experience, issues and trends. Ecol Econ 31: 39-62.
